# Systemic lupus erythematosus and rheumatoid arthritis with lymphatic system involvement: a study based on non-contrast MR lymphangiography and ^99^TC^m^-DX lymphoscintigraphy

**DOI:** 10.1186/s12880-026-02247-w

**Published:** 2026-02-28

**Authors:** Lei Yang, Yuguang Sun, Zhe Wen, Rengui Wang, Yunlong Yue

**Affiliations:** 1 Department of Medical Image, Beijing Shijitan Hospital, Capital Medical University, Yangfangdian Tieyi Road No.10, Haidian District, Beijing, 100038 China; 2 Department of Lymph Surgery, Beijing Shijitan Hospital, Capital Medical University, Yangfangdian Tieyi Road No.10, Haidian District, Beijing, 100038 China; 3 Department of Nuclear Medicine, Beijing Shijitan Hospital, Capital Medical University, Yangfangdian Tieyi Road No.10, Haidian District, Beijing, 100038 China

**Keywords:** Systemic lupus erythematosus, Rheumatoid arthritis, Lymphatic system, Non-contrast MR lymphangiography, ^99^TC^m^-DX lymphoscintigraphy

## Abstract

**Objective:**

To investigate the diagnostic value of combined non-contrast MR lymphangiography (NCMRL) and ^99^TC^m^-DX lymphoscintigraphy in systemic lupus erythematosus(SLE) and rheumatoid arthritis(RA) with lymphatic system involvement.

**Methods:**

A retrospective analysis was conducted on the clinical and imaging data of 25 SLE patients with lymphatic system involvement and 18 RA patients with lymphatic system involvement. All patients underwent NCMRL and ^99^TC^m^-DX lymphoscintigraphy examinations. The chi-square test or Fisher’s exact test was used to analyze the distribution differences of abnormal lymphatic system signs in NCMRL and ⁹⁹Tcᵐ-DX lymphangiography between the two groups of patients. McNemar’s test was used to compare the abnormal lymphatic vessel signs detected by the two imaging modalities in 43 patients with SLE or RA with lymphatic system involvement. The Kappa test was applied to evaluate the diagnostic consistency of the two imaging modalities in identifying abnormal lymphatic vessels in these patients. *P* < 0.05 was statistically significant.

**Results:**

MRL showed that the incidence of thoracic duct outlet obstruction in SLE patients with lymphatic system involvement (25, 100.0%) was significantly higher than that in RA patients with lymphatic system involvement (6, 33.3%) (*P* = 0.000). In ⁹⁹Tcᵐ - DX lymphoscintigraphy, the incidence of chylous effusion in SLE patients with lymphatic system involvement (22, 88.0%) was significantly higher than that in RA patients with lymphatic system involvement (4, 22.2%), while the incidence of limb lymphedema in RA patients with lymphatic system involvement (14, 77.8%) was significantly higher than that in SLE patients with lymphatic system involvement (4 cases, 20.0%) (*P* = 0.000). There was no statistically significant difference (*P* > 0.05) in the detection rates of obstruction of the thoracic duct outlet, tortuous dilation of the thoracic segment of the thoracic duct, abnormality of the bronchial mediastinum, and dilation of the iliac lymphatic vessels and lumbar trunk between the two examinations. The Kappa values were rated as follows: almost perfect (1.000), good (0.788), moderate (0.482), and fair agreement (0.256).

**Conclusion:**

⁹⁹Tcᵐ-DX lymphoscintigraphy dynamically reflects lymphatic reflux and identifies lymphedema and chylous effusion in SLE and RA patients with lymphatic involvement, but has low spatial resolution. NCMRL clearly displays systemic lymphatic vessel abnormalities and surrounding tissue conditions, yet cannot confirm chylous effusions or localize active chyle leakage. Combined use of the two modalities provides comprehensive imaging information for the clinical diagnosis of SLE and RA with lymphatic system involvement.

## Introduction

Systemic lupus erythematosus (SLE) and rheumatoid arthritis (RA) are two important subtypes of connective tissue diseases [1], which are characterized by the immune system attacking the body’s own connective tissue. SLE can damage various organ systems, including the central nervous system, kidneys, cardiovascular system, and hematopoietic system [2]. RA primarily affects the joints, leading to synovitis and hyperplasia, and causing joint pain, swelling, and injury [3]. The progression of RA can lead to systemic involvement and multiple organ dysfunction. Lymphatic system involvement is a rare complication of SLE and RA. Inflammatory mediators in patients with SLE and RA can alter the permeability of lymphatic vessel walls and the function of lymphatic pumps [4], leading to impaired lymphatic drainage. Clinically, this condition manifests as lymphedema and chylous effusion, which adversely affect patients’ quality of life [5, 6]. Existing research demonstrates that there are differences in the clinical manifestations of lymphatic system involvement between SLE and RA: SLE with lymphatic system involvement is mainly characterized by chylous effusion, and limb lymphedema is only seen in individual cases [7, 8]; RA with lymphatic system involvement manifests as limb lymphedema [9]. The development of chylous effusion in patients with SLE may be closely associated with thoracic duct obstruction [10]. Abnormal fibrinolytic mechanisms in RA patients may lead to lymphatic vessel obstruction, which could be one of the causes of lymphedema in RA [11]. Schwartz N et al. suggested that clarifying the etiology and clinical manifestations of lymphatic dysfunction in these autoimmune diseases may help better target upstream mediators, and could also reveal that targeting the lymphatic system is one of the mechanisms of action for certain drugs; meanwhile, the improvement of lymphatic function should be incorporated as part of disease treatment [12].

At present, research reports on SLE and RA with Lymphatic system involvement remain limited, and clinical cases of single diseases are mainly reported. There is no relevant study on the characteristics of NCMRL and ^99^TC^m^-DX lymphoscintigraphy for these two diseases. Nuclide lymphatic imaging is currently the “gold standard” for diagnosing lymphatic reflux disorders [13]. Magnetic resonance lymphatic imaging has the advantage of accurately displaying abnormal morphology and structure of the lymphatic system [14]. Both techniques have their own characteristics in the diagnosis of lymphatic reflux disorders. The aim of this study is to use NCMRL and ^99^TC^m^-DX lymphoscintigraphy to analyze the differences in imaging manifestations of SLE and RA with lymphatic system involvement, and to explore the diagnostic value of these two imaging modalities for SLE and RA with lymphatic system involvement.

## Materials and methods

### Patients

A retrospective collection of clinical data from 188 patients with highly suspected lymphatic system involvement in Beijing Shijitan Hospital between June 2014 and October 2024 was conducted, including 85 cases of SLE and 103 cases of RA. Suspected manifestations of lymphatic system involvement included: (1) Refractory serous effusion: no significant reduction or recurrent episodes of serous effusion after standardized treatment for SLE/RA underlying diseases; drainage fluid appearing white, milky, yellow, or chylous, with suspicion of chylous effusion. (2) Persistent edema lasting ≥ 1 month after excluding common etiologies such as cardiac, renal, hepatic, hypoproteinemia, or venous thrombosis, with evidence of abnormal lymphatic circulation in the edematous area, including skin thickening, roughness, non-pitting changes, or recurrent skin infections. All patients underwent lymphatic imaging with one or more of the following lymphatic imaging: direct lymphangiography, computed tomography lymphangiography, NCMRL or ⁹⁹Tcᵐ-DX lymphoscintigraphy. A total of 101 patients were ultimately diagnosed with lymphatic system involvement via lymphatic imaging, including 48 SLE patients and 53 RA patients.

**Fig. 1:**
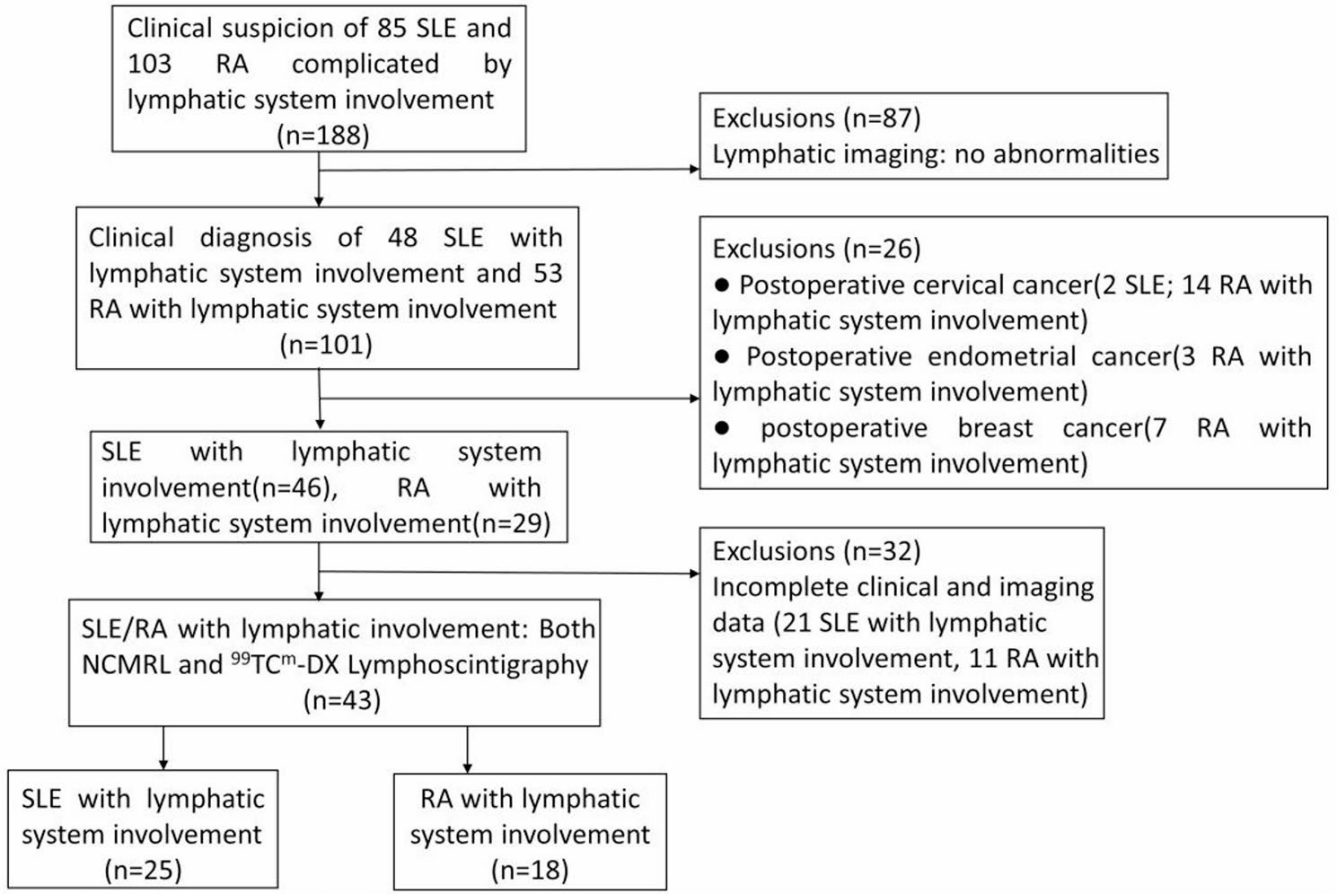
Flow chart of study population inclusion. 188 patients with SLE and RA with lymphatic system involvement were included between June 2014 and October 2024, 145 patients were excluded and finally 43 patients were included in the study

The inclusion criteria were as follows: (1) Between 2014 and 2019, the diagnosis of SLE was based on the classification criteria proposed by the Systemic Lupus International Collaborating Clinics in 2012 [15]. After 2019, the diagnosis of SLE has been based on the 2019 EULAR/ACR Classification Criteria [16]. RA was diagnosed according to the 2010 EULAR/ACR RA classification criteria [17], and (2) underwent NCMRL and ^99^TC^m^-DX lymphoscintigraphy examinations, and showed abnormal manifestations of lymphatic system involvement, such as chylous fluid or lymphedema; none of the included patients had a history of lymphatic system-related surgery before undergoing their first NCMRL and ⁹⁹Tcᵐ-DX lymphoscintigraphy. The diagnostic criteria for chylous fluid required at least one of the following: a positive chyle test result, a triglyceride concentration in serosal fluid > 110 mg/dL, or lymphoscintigraphy showing radioactive uptake in the serosal cavity [18]. Lymphedema is defined as the abnormal accumulation of protein-rich interstitial fluid [19]. In this study, all patients with limb lymphedema were diagnosed by ^99^TC^m^-DX lymphoscintigraphy, showing radiopharmaceutical retention in the subcutaneous tissues of the affected limbs.The exclusion criteria included patients who had not undergone both NCMRL and ^99^TC^m^-DX lymphoscintigraphy, as well as those with lymphatic involvement caused by concomitant tumours, surgery, or infections (such as filariasis, tuberculosis, etc.). A total of 145 patients were excluded from this study: 87 of these patients demonstrated no abnormal findings on imaging examinations for lymphatic system involvement; among the 101 patients diagnosed with lymphatic system involvement, 58 were excluded for failing to meet the inclusion criteria (26 were post-tumor resection patients and 32 had incomplete clinical and imaging data). Finally, 43 patients were enrolled in the study, including 25 cases of SLE with lymphatic system involvement and 18 cases of RA with lymphatic system involvement. The inclusion and exclusion criteria are shown in Fig. 1. Among the 25 SLE patients with lymphatic system involvement, all were female. The median age of SLE onset was 29.0 (interquartile range, IQR: 24.5, 51.0) years (range, 19–72 years), and the median duration of SLE was 72 (IQR: 22,108) months (range, 10–360 months). The average age of lymphatic system involvement in patients with SLE was 42.6 ± 15.3 years (range 20–75 years), and the median duration of lymphatic system involvement in patients with SLE was 11 (IQR: 3,21) months (range, 1–48 months). Among the 18 RA patients with lymphatic system involvement, there were 16 females and 2 males. The average age of RA onset was 42.8 ± 11.9 years (range, 22–60 years), and the median duration of RA was 180 (IQR: 120,360) months (range, 6–516 months). The average age of lymphatic system involvement in patients with RA was 60.3 ± 9.6 years (range, 37–71 years), and the median duration of lymphatic system involvement in patients with RA was 12 (IQR: 7.5,39) months (range, 5–120 months). Five SLE patients (20.0%) were diagnosed with protein-losing enteropathy by ^99^Tc^m^-labelled human serum albumin (^99^Tc^m^-HSA) scintigraphy, and seven SLE patients (28.0%) had lupus nephritis. The demographic characteristics and clinical features of the SLE and RA patients with lymphatic system involvement are shown in Table 1.

**Table 1:** The demographic characteristics and clinical features of the SLE and RA patients with lymphatic system involvement

Variable	SLE with lymphatic system involvement (*n* = 25)	RA with lymphatic system involvement (*n* = 18)	*P* value
SLE/RA onset age, yrs	29(24.5,51.0)	42.8 ± 11.9	0.089
Disease duration of SLE/RA, mos	**72(22**,**108)**	**180(120**,**360)**	**0.000**
Lymphatic system involvement onset age, yrs	**42.6 ± 15.3**	**60.3 ± 9.6**	**0.000**
Disease duration of lymphatic system involvement, mos	11(3,21)	12(7.5,39)	0.100
Chest tightness	**15(60.0)**	**2(11.1)**	**0.001**
Fever	2(8.0)	0(0.0)	0.502
Abdominal distension	5(20.0)	2(11.1)	0.680
Diarrhea	2(8.0)	0(0.0)	0.502
Mucocutaneous involvement	**17(68.0)**	**0(0.0)**	**0.000**
Dry mouth and eyes	3(12.0)	1(5.6)	0.628
Raynaud’s phenomenon	3(12.0)	1(5.6)	0.628
Limb edema	**7(28.0)**	**14(77.8)**	**0.002**
Arthritis	**3(12.0)**	**18(100.0)**	**0.000**
Hypoproteinemia	**15(60.0)**	**0(0.0)**	**0.000**

The values with statistical significance (*P* < 0.05) are displayed in bold

In this study, 18 patients underwent thoracic duct surgery. Among the SLE patients with lymphatic system involvement, 2 underwent thoracic duct compression band release surgery, and 12 underwent thoracic duct terminal adhesion release surgery. Among patients with RA combined with lymphatic system involvement, 4 underwent surgical release of adhesions at the terminal segment of the thoracic duct. Intraoperative exploration of the jugular venous angle revealed numerous dense cellulose adhesions and collapsed lymphatic vessel lumina, which obstructed the pathway for lymphatic fluid flow back into the bloodstream.

This retrospective study was approved by the Ethics Committee of the Beijing Century Altar Hospital affiliated with Capital Medical University. Owing to the retrospective nature of the study, the requirement for informed consent to access medical records and imaging data was waived.

## Non-contrast MR lymphangiography (NCMRL)

NCMRL was performed with a Philips Ingenia 3.0T MR scanner using a head coil combined with a body coil, and a 3D T2-weighted water imaging sequence. Scanning parameters included a repetition time (TR) of 2500–3000 milliseconds, an echo time (TE) of 550–600 milliseconds, an echo chain length of 85–105, a scanning range of 36 cm × 30 cm × 9 cm, and a voxel width of 10 mm × 10 mm × 10 mm. Ninety acquisition slices were obtained. Two sections were collected, covering the area from the neck base to the pelvic floor level. Post-processing was performed using maximum density projection (MIP) technology to obtain NCMRL images. Non-contrast abdominal and pelvic MRI scan using a body coil. For abdominal MRI, the scanning parameters were set as follows: axial T2-weighted imaging(T2WI) with TR/TE of 3000/110 ms, matrix of 268 × 253, slice thickness of 4 mm and field of view (FOV) of 400 × 320 mm; diffusion-weighted imaging (DWI) with TR/TE of 3659/84 ms, matrix of 180 × 150, FOV of 400 × 320 mm and b-values of 0, 800 s/mm²; and axial T1-weighted imaging (T1WI)-mDIXON with TR/TE of 5.8/1.8 ms, matrix of 224 × 175, slice thickness of 3 mm and FOV of 400 × 320 mm; for pelvic MRI, the scanning parameters were configured as axial T2WI with TR/TE of 3000/110 ms, matrix of 268 × 253, slice thickness of 4 mm and FOV of 240 × 240 mm; DWI with TR/TE of 3659/84 ms, matrix of 144 × 110, FOV of 400 × 300 mm and b-values of 0, 1000 s/mm²; and axial T1WI-mDIXON with TR/TE of 5.8/1.8 ms, matrix of 224 × 175, slice thickness of 3 mm and FOV of 400 × 317 mm.

^**99**^**TC**^**m**^**-DX lymphoscintigraphy**.

Dual-head single-photon emission computed tomography (SPECT, Symbia T16; Siemens Medical Systems) equipped with a low-energy high-resolution collimator was used. The photopeak was set at 141 keV with a window width of 20%, the acquisition speed was 14–18 cm/min, the acquisition matrix was 512 × 512 with an acquisition zoom factor of 1.0. The radiotracer used was ^99^TC^m^-DX with a labeling efficiency of > 95%. For whole-body imaging, an equal dose of ^99^TC^m^-DX (111–185 MBq, 0.10–0.15 mL per injection site) was slowly administered via subcutaneous injection into the 1st and 4th interdigital spaces of both feet, with injection intervals < 1 min. After 5 min of injection, patients were instructed to walk on the ground; for those unable to walk, lower limb flexion-extension exercises were performed instead. Whole-body images were acquired at 10 min, 1 h, 3 h, and 6 h post-injection, respectively. For patients with upper limb edema, ^99^TC^m^-DX (185 MBq, 0.15 mL per limb) was subcutaneously injected into the interdigital webs of both hands. After injection, patients were advised to perform hand movements to facilitate radiotracer drainage. The scanning range extended continuously from the hand injection sites to the level of the pubic symphysis, covering both hands, upper extremities, axillary fossa, chest wall, and mediastinal region. Images were obtained at 10 min, 1 h, 3 h, and 6 h post-injection, respectively. Images acquired at 10 min and 1 h post-injection were defined as early-phase imaging, those at 3 h as intermediate-phase imaging, and those at 6 h as delayed-phase imaging.

## Imaging analysis

Two radiologists with more than 10 years of clinical experience independently reviewed the images. Before image review, the radiologists were kept unaware of the patients’ clinical information and only performed analyses based on the imaging data. If discrepancies arose between the two radiologists, a third senior radiologist with over 15 years of clinical experience was consulted to participate in the discussion to reach a consensus.

Analysis of lymphatic vessel abnormalities on NCMRL revealed the following findings: (1) Abnormalities in the thoracic duct, iliac lymphatic vessels, and lumbar trunk: variation, dilation or stenosis of the thoracic duct lumen, multiple tortuous and small lumina were observed in the jugular venous angle, indicating obstruction of the thoracic duct outlet; and bead-like, reticular, or cystic T2-weighted high signal around the iliac vessels and retroperitoneal large vessels indicating obstruction of lymphatic reflux leading to iliac lymphatic vessels and lumbar trunk dilation. (2) Abnormal lymphatic reflux: abnormal display of the bronchial mediastinal trunk, subclavian trunk, and cervical trunk, manifested as tortuous and widened tubular, spotted or quasicircular abnormally high signals on T2-weighted imaging. Analysis of abnormal findings on MRI of the chest, abdomen, and pelvic demonstrated the following: atelectasis, pleural effusion, mediastinal fat space opacification, pericardial effusion, abdominal and pelvic effusions, subcutaneous edema involving the chest, abdominal, and pelvic walls, and intestinal and mesenteric abnormalities.

Evaluation of ^99^TC^m^-DX lymphoscintigraphy findings included the following: (1) whether the lymphatic vessels and lymph node chains in the upper and lower limbs were intact and whether the lymphatic return was unobstructed. Normal lymphatic reflux: imaging 6 h after injection revealed no radioactive retention in the lymphatic vessels of the upper or lower limbs. The axillary lymph nodes and subclavian lymph nodes (inguinal lymph nodes, iliac lymph nodes, and lumbar lymph nodes) were round or oval in shape with clear boundaries. Slow lymphatic reflux was defined as follows: After 6 h of injection of contrast agent, imaging showed radioactive retention in the lymphatic vessels of the upper (lower) limbs, with normal, few, or faint lymph node chains. Non-reflux type: the contrast agent remained at the injection site, and the affected side’s lymphatic vessels and nodes were not visible; the presence of abnormal distribution of radioactivity in the subcutaneous tissue of the limbs or trunk was judged as lymphedema; (2) the radioactive distribution of the thoracic duct at the jugular venous angle could be divided into three types: Type I was abnormally concentrated, with a persistent radioactive concentration of contrast agent at the left jugular venous angle, indicating obstruction of the thoracic duct outlet; Type II was the ectopic drainage type (including bilateral jugular venous angle drainage of the thoracic duct, and right jugular venous angle drainage of the thoracic duct), with continuous radioactive concentration of contrast agent at the bilateral or right jugular venous angle, indicated variation and outlet obstruction of the thoracic duct; and Type III showed no or transient visualization at the left jugular venous angle, indicating no obstruction at the the thoracic duct outlet [20] .Thoracic duct-related tracer accumulation manifests as cord-like or irregular dense opacities, which are distributed along the course of the thoracic duct and extend to the jugular venous angle. If radioactive tracer uptake occurs in the cervical lymph nodes, it presents as round or oval radioactivity-dense foci with clear boundaries, consistent with the distribution of the surrounding lymph node chains. (3) Whether there was abnormal tracer reflux in the subclavian trunks, jugular trunks, or bronchomediastinal trunks; whether there was dilatation and tortuosity in the iliac and lumbar lymphatic chains (mainly referring to the iliac lymphatic vessels and lumbar trunks), which was manifested as enlarged, clustered, or beaded hyperradioactive foci distributed along the course of the iliac lymphatic vessels and lumbar trunks. (4) Whether there was radioactive tracer accumulation in the thoracic cavity, abdominal cavity, or pericardium, wherein abnormally increased radioactivity in these respective regions was defined as chylothorax, chylous ascites, or chylopericardium.

### Statistical analysis

Statistical analysis was performed using SPSS 26.0 software (SPSS, version 26.0; IBM, Armonk, NY, USA). Metric data that conformed to a normal distribution were expressed as mean ± standard deviation. In contrast, metric data that did not conform to a normal distribution are represented by the median (interquartile range). Non-normally distributed metric data were evaluated using the Mann‒Whitney U test, whereas normally distributed metric data were assessed using the independent samples t test. If the P value was less than 0.05, the difference between the two groups was considered significant. Comparisons of qualitative data between groups were conducted using the chi-square test or Fisher’s exact test, and *P* < 0.05 was considered statistically significant. McNemar’s test was used to compare the lymphatic vessel abnormalities detected by NCMRL and ^99^TC^m^-DX lymphoscintigraphy in 43 SLE and RA patients. The difference was considered to be statistically significant when *P* < 0.05. The kappa test was used to evaluate the consistency of NCMRL and ^99^TC^m^-DX lymphoscintigraphy in observing lymphatic vessel abnormalities in 43 SLE and RA patients with lymphatic system involvement. The kappa values were rated as follows: minor (less than 0.20), fair (0.21–0.40), moderate (0.41–0.60), good consistency (0.61–0.80), and almost perfect agreement (0.81–1.00).

## Results

### Abnormal manifestations on chest and abdominal MRI

The incidence of atelectasis was 76.0% (19/25), and that of concurrent pleural and abdominal effusions was 56.0% (14/25) in SLE patients with lymphatic system involvement; in RA patients with lymphatic system involvement, the incidence of atelectasis was 22.2% (4/18), and that of concurrent pleural and abdominal effusions was 0% (0/18). The incidence of atelectasis, pleural and abdominal effusions was significantly higher in SLE patients with lymphatic system involvement than in RA patients with lymphatic system involvement (*P* = 0.000) (Table 2).

**Table 2:** Abnormal MRI manifestations in patients with SLE and RA with lymphatic system involvement

Abnormal manifestations on MRI	SLE with lymphatic system involvement (*n* = 25)	RA with lymphatic system involvement (*n* = 18)	*P* value
Atelectasis	**19(76.0)**	**4(22.2)**	**0.000**
Turbidity of mediastinal fat space	3(12.0)	0(0.0)	0.252
Pericardial effusion	4(16.0)	0(0.0)	0.127
Diffuse thickening of the small intestine wall	3(12.0)	0(0.0)	0.252
Turbidity of mesenteric fat space	5(20.0)	0(0.0)	0.064
Pleural effusion	5(20.0)	4(22.2)	1.000
Ascites	2(8.0)	3(16.7)	0.634
Pleural and peritoneal effusions	**14(56.0)**	**0(0.0)**	**0.000**
Subcutaneous edema of chest wall	3(12.0)	1(5.6)	0.628
Subcutaneous edema of abdominal wall	1(4.0)	1(5.6)	1.000
Subcutaneous edema of chest and abdominal walls	5(20.0)	0(0.0)	0.064

The values with statistical significance (*P* < 0.05) are displayed in bold

## NCMRL abnormal manifestations

**Fig. 2:**
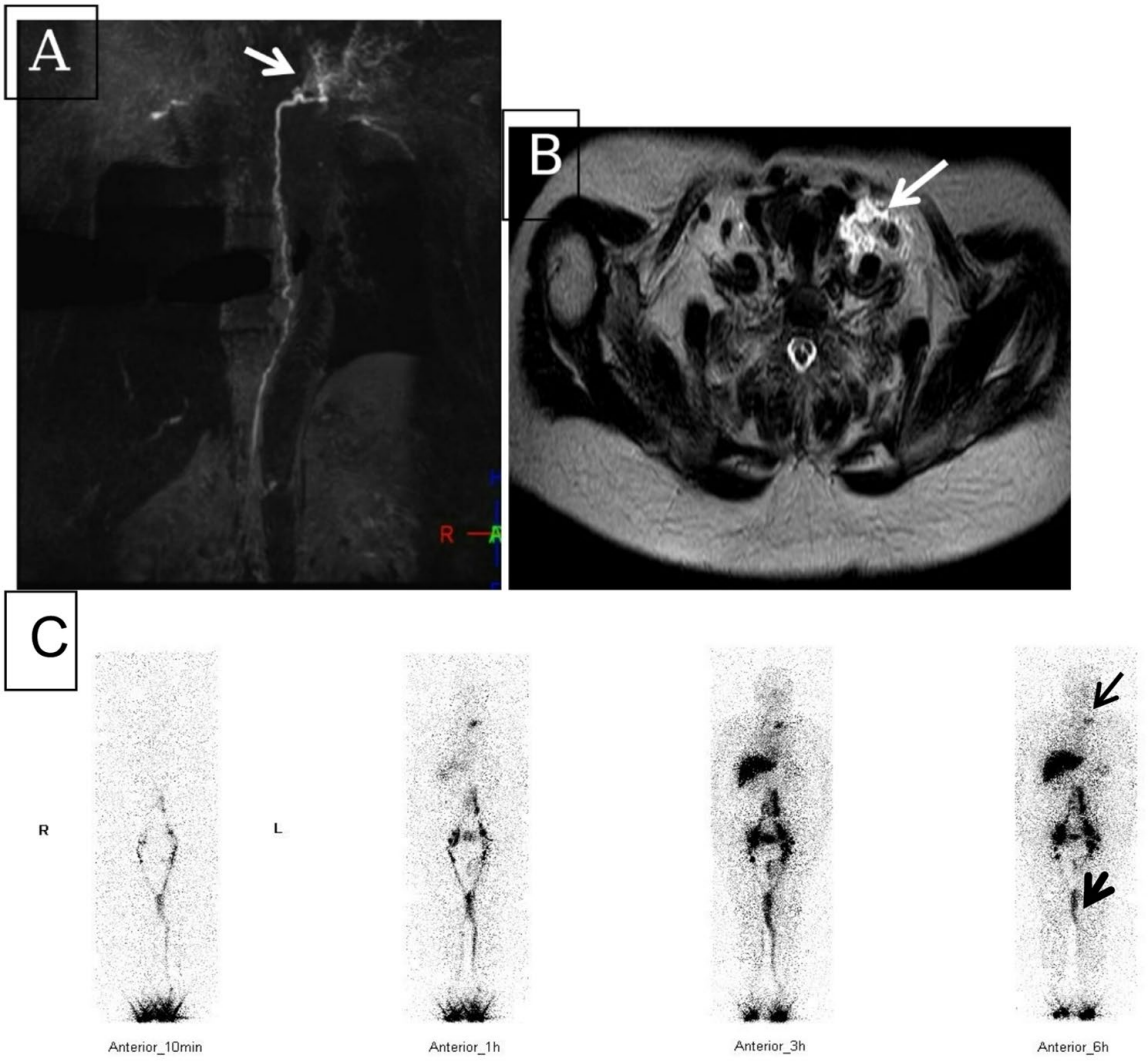
**A**–**C** A patient with RA with lymphatic system involvement. (**A**) Coronal Non-contrast Magnetic Resonance Lymphangiography (NCMRL) showing multiple abnormal small lymphatic vessels (white arrow) in the left jugular venous angle; (**B**) Axial NCMRL showing multiple abnormal small lymphatic vessels (white arrow) in the left jugular venous angle. (**C**) ^99^TC^m^-DX lymphoscintigraphy (anterior): continuous visualization of the left venous angle at 1 h, 3 h, and 6 h (thin black arrow); a small amount of contrast agent is retained subcutaneously on the inner side of the left thigh (thick black arrow), indicating lymphedema

**Fig. 3:**
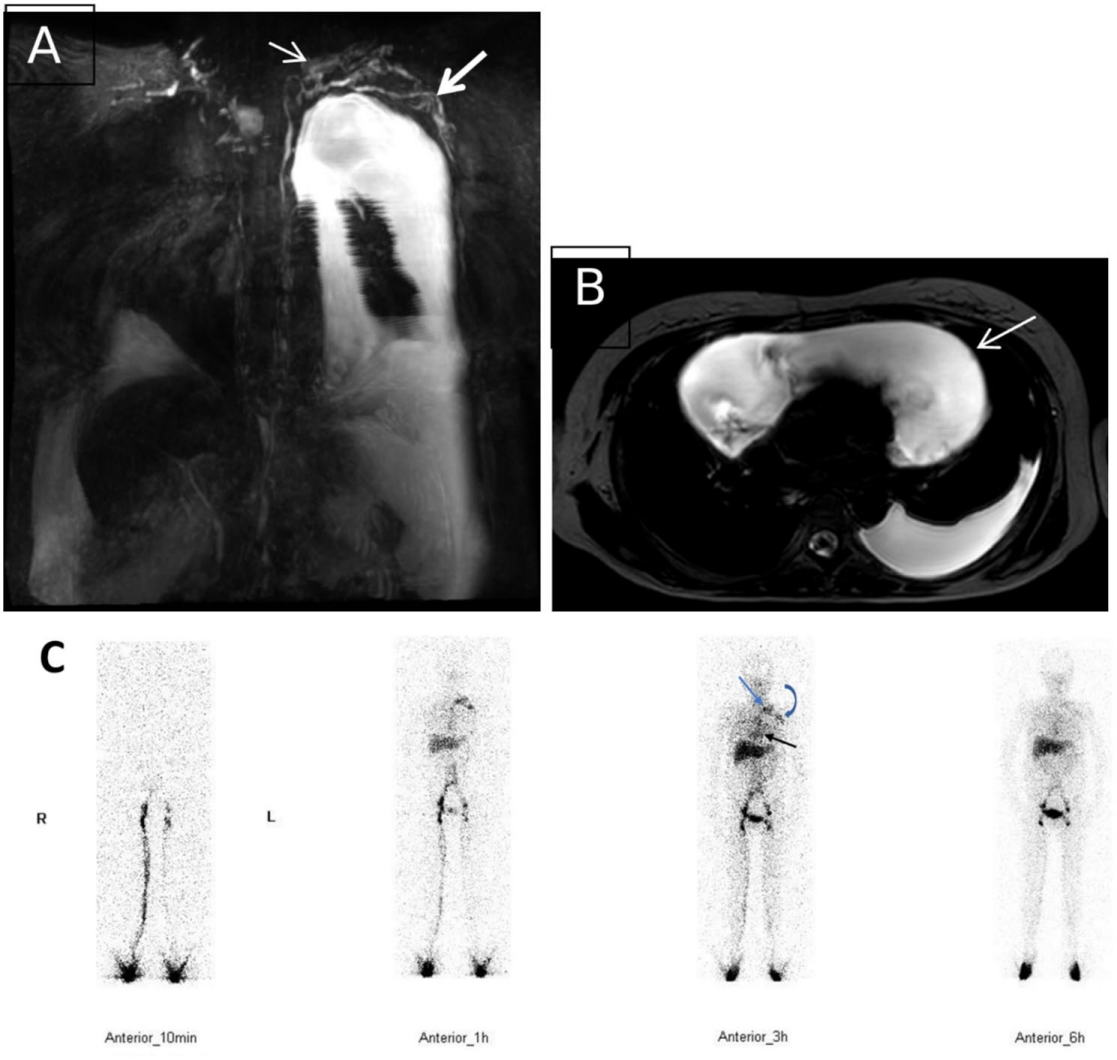
**A**–**C** A patient with SLE with lymphatic system involvement. (**A**) Coronal NCMRL image showing multiple abnormal lymphatic vessels in the left jugular venous angle; the left subclavian trunk can also be observed. (**B**) Coronal NCMRL showing pericardial effusion. (**C**) ^99^TC^m^-DX lymphoscintigraphy (anterior position) showing abnormal concentration in the left jugular venous angle (blue arrow) and abnormal display of the left subclavian trunk (blue curved arrow), indicating thoracic duct outlet obstruction with left subclavian trunk lymphatic reflux; radioactive filling of the pericardium(black arrow), indicating chylopericardium

**Fig. 4:**
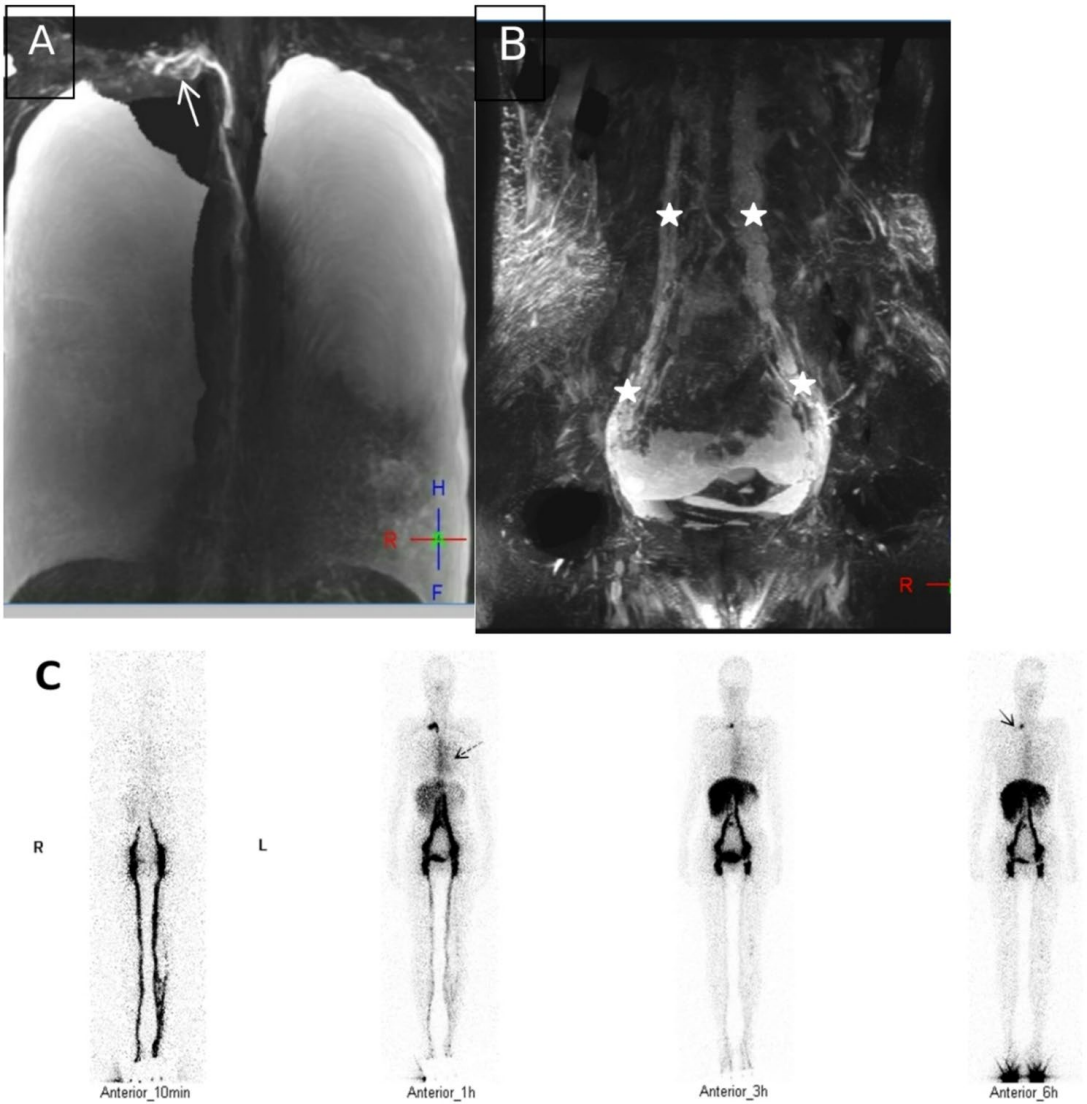
**A**–**C** A patient with SLE with lymphatic system involvement. (**A**) Coronary NCMRL showing drainage of the thoracic duct to the right jugular venous angle (white arrow). (**B**) Coronary NCMRL showing widened T2-weighted hyperintensities (pentagrams) around the bilateral retroperitoneal large vessels and iliac vessels. (**C**) ^99^TC^m^-DX lymphoscintigraphy (anterior): right jugular venous angle is continuously visible at 1 h, 3 h, and 6 h (black arrow); elevated radioactivity in the left chest cavity (dashed black arrow) indicates left chylous pleural effusion

**Fig. 5:**
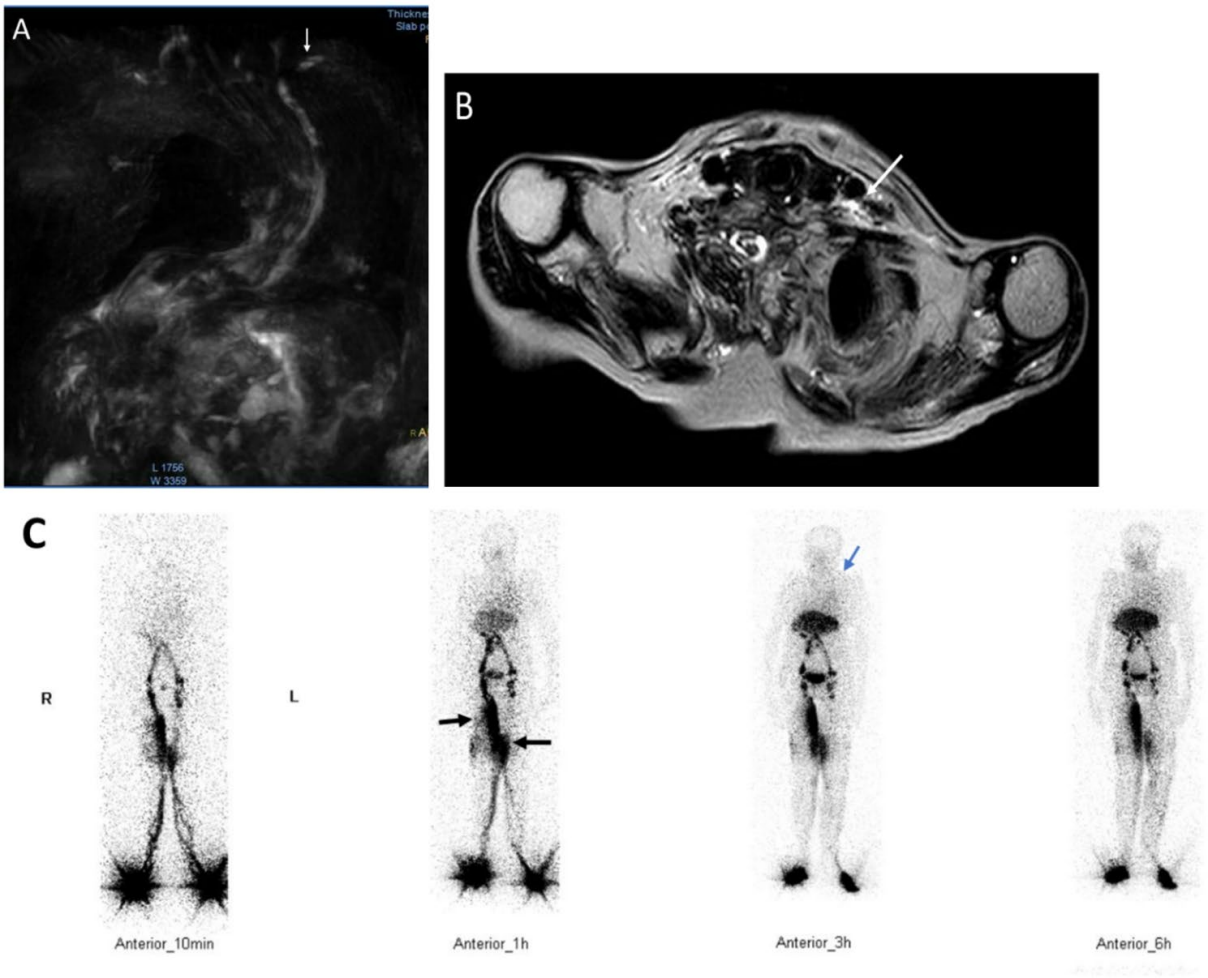
**A**–**C** A patient with RA with lymphatic system involvement and scoliosis of the spine. (**A**) Coronary NCMRL and (**B**) Axial NCMRL showing no stenosis of the thoracic duct lumen at the left venous angle region, and no abnormally tortuous tubular structures in the surrounding area(white arrow). (**C**) ^99^TC^m^-DX lymphoscintigraphy (anterior): no contrast agent enhancement at the venous angle (blue arrow), indicating no obstruction at the thoracic duct outle; contrast agent retention in the subcutaneous tissue of bilateral lower limbs (black arrows), suggesting lymphedema

^**99**^**TC**^**m**^**-DX lymphoscintigraphy**.

In NCMRL examination, the incidence of thoracic duct outlet obstruction (including the thoracic duct outlet in the left jugular venous angle, in the right jugular venous angle, and in the bilateral jugular venous angle) was 100.0% (25/25) in SLE patients with lymphatic system involvement, compared with 33.3% (6/18) in RA patients with lymphatic system involvement. The incidence of thoracic duct outlet obstruction was significantly higher in SLE patients with lymphatic system involvement than in RA patients with lymphatic system involvement (*P* = 0.000) (Table 3; Figs. 2A-B, 3A-B, 4A-B and 5A-B).

**Table 3:** **NC**MRL manifestations of SLE and RA patients with lymphatic system involvement

Abnormal manifestations on NCMRL	SLE with lymphatic system involvement (*n* = 25)	RA with lymphatic system involvement (*n* = 18)	*P* value
Obstruction of the thoracic duct outlet	**25(100.0)**	**6(33.3)**	**0.000**
Obstruction of the thoracic duct outlet at the left jugular vein angle	**23(92.0)**	**6(33.3)**	**0.000**
Obstruction of the thoracic duct outlet at the right jugular vein angle	1(4.0)	0(0.0)	1.000
Bilateral jugular vein angle drainage of the thoracic duct and obstruction of the outlet	1(4.0)	0(0.0)	1.000
Tortuosity and dilation of the thoracic segment of thoracic duct	2(8.0)	0(0.0)	0.502
Abnormal visualisation of the subclavian trunk	7(28.0)	1(5.6)	0.111
Abnormal visualisation of the bronchial mediastinal trunk	3(12.0)	0(0.0)	0.252
Dilation of iliac lymphatic vessels and lumbar trunks	5(20.0)	1(5.6)	0.375

The values with statistical significance (*P* < 0.05) are displayed in bold

All 25 (100.0%) of the SLE patients with lymphatic system involvement had obstruction of the thoracic duct outlet. Among these patients, 23 (92.0%) had continuous visualization of at the left jugular venous angle, 1 patient (4.0%) showed continuous visualization of the bilateral jugular venous angle (indicating bilateral drainage of the thoracic duct with outlet obstruction), and 1 patient (4.0%) showed continuous visualization of the right jugular venous angle but no visualization of the left venous angle (indicating right drainage of the thoracic duct with outlet obstruction); 22 patients (88.0%) with lymphatic system involvement were found to have chylous effusion: 12 patients (48.0%) exhibited chylous pleural effusion, 1 patient (4.0%) had chylous peritoneal effusion, 6 patients (24.0%) had chylous pleural effusion with peritoneal effusion, 2 patients (8.0%) had chylous pericardial effusion, and 1 patient (4.0%) had chylous pericardial effusion with pleural effusion.

Among patients with RA with lymphatic system involvement, 12 (66.7%) had no obstruction at the thoracic duct outlet, which manifested as no visualization or visualization only at 1 h at the left jugular venous angle. All 12 of these 12 patients (66.7%) with RA also presented with limb lymphoedema: 1 (5.6%) with unilateral upper limb lymphoedema, 3 (16.7%) with bilateral upper limb lymphoedema, 4 (22.2%) with unilateral lower limb lymphoedema, and 4 (22.2%) with bilateral lower limb lymphoedema. The remaining 6 patients (33.3%) with RA developed thoracic duct outlet obstruction, which manifested as persistent visualization or widening of the left jugular vein angle. Among these 6 RA patients (33.3%), 2 (11.1%) had unilateral lower limb lymphoedema, 2 (11.1%) had chylous pleural effusion, and 2 (11.1%) had chylous peritoneal effusion. The incidence of chylous effusion was 88.0% (22/25), and that of continuous visualization of left jugular venous angle was 92.0% (23/25) in SLE patients with lymphatic system involvement; in RA patients with lymphatic system involvement, the incidence of chylous effusion was 22.2% (4/18) and that of continuous visualization of left jugular venous angle was 33.3% (6/18). The incidence of chylous effusion and persistent visualization of the left jugular venous angle was significantly higher in SLE patients with lymphatic system involvement than in RA patients with lymphatic system involvement (*P* = 0.000). The incidence of limb lymphedema (including unilateral or bilateral limbs, and upper or lower limbs) was 77.8% (14/18) in RA patients with lymphatic system involvement, compared with 16.0% (4/25) in SLE patients with lymphatic system involvement. The incidence of limb lymphedema was significantly higher in RA patients with lymphatic system involvement than in SLE patients with lymphatic system involvement (*P* = 0.000) (Table 4; Figs. 2C, 3C and 4C, and 5C).

**Table 4:** ^99^TC^m^-DX Lymphoscintigraphic manifestations in SLE and RA Patients with Lymphatic System Involvement

^99^TC^m^-DX Lymphoscintigraphic manifestations	SLE with lymphatic system involvement (*n* = 25)	RA with lymphatic system involvement (*n* = 18)	*P* value
No visualization or visualization at 1 h of the left jugular venous angle	**0(0.0)**	**12(66.7)**	**0.000**
Continuous visualization of bilateral jugular venous angles	1(4.0)	0(0.0)	1.000
Continuous visualization of left jugular venous angle	**23(92.0)**	**6(33.3)**	**0.000**
Continuous visualization of the right venous angle, no visualization of the left venous angle	1(4.0)	0(0.0)	1.000
Dilation and persistent visualization of the thoracic segment of thoracic duct	2(8.0)	1(5.6)	1.000
Radiographic contrast reflux of left subclavian trunk	2(8.0)	0(0.0)	0.502
Radiographic contrast reflux of bronchial mediastinal trunk	1(4.0)	0(0.0)	1.000
Dilation of iliac lymphatic vessels and lumbar trunks	1(4.0)	0(0.0)	1.000
Limb lymphedema (unilateral/bilateral, upper/lower limb)	**4(16.0)**	**14(77.8)**	**0.000**
Unilateral upper limb lymphedema	0(0.0)	1(5.6)	0.419
No lymphatic reflux in the edematous upper limb; slow lymphatic return in the contralateral upper limb	0(0.0)	1(5.6)	0.419
Bilateral upper limb lymphedema	0(0.0)	3(16.7)	0.066
No lymphatic reflux in both upper limbs	0(0.0)	2(11.1)	0.169
Slow lymphatic reflux in both upper limbs	0(0.0)	1(5.6)	0.419
Unilateral lower limb lymphedema	3(12.0)	6(33.3)	0.133
No lymphatic reflux in the edematous lower limb; slow lymphatic return in the contralateral lower limb	1(4.0)	2(11.1)	0.562
No lymphatic reflux in the edematous lower limb; normal lymphatic reflux in the contralateral lower limb	0(0.0)	3(16.7)	0.066
Slow lymphatic return in the edematous lower limb; normal lymphatic reflux in the contralateral lower limb	2(8.0)	1(5.6)	1.000
Bilateral lower limb lymphedema	1(4.0)	4(22.2)	0.144
No lymphatic reflux in the edematous lower limb; slow lymphatic return in the contralateral lower limb	0(0.0)	1(5.6)	0.419
Slow lymphatic reflux in both lower limbs	1(4.0)	3(16.7)	0.293
No lymphedema in the both lower limbs, slow lymphatic return	**14(56.0)**	**1(5.6)**	**0.001**
chylous effusion (pleural/peritoneal/pericardial)	**22(88.0)**	**4(22.2)**	**0.000**
Chylous hydrothorax	**12(48.0)**	**2(11.1)**	**0.011**
Chylous ascites	1(4.0)	2(11.1)	0.562
Chylous pleural and peritoneal effusions	**6(24.0)**	**0(0.0)**	**0.032**
Chylopericardium	2(8.0)	0(0.0)	0.502
Chylopericardium and chylous hydrothorax	1(4.0)	0(0.0)	1.000

The values with statistical significance (*P* < 0.05) are displayed in bold

## Comparison of imaging features between NCMRL and ^99^TC^m^-DX lymphoscintigraphy

The Kappa values for the diagnostic agreement between NCMRL and ^99^TC^m^-DX lymphoscintigraphy across different lymphatic system abnormalities, along with the corresponding agreement levels, were as follows: (1) Obstruction at the thoracic duct outlet (left jugulovenous angle): Kappa = 1.000 (almost perfect agreement); (2) Obstruction at the right jugulovenous drainage outlet of the thoracic duct: Kappa = 1.000 (almost perfect agreement); (3) Obstruction at the bilateral jugulovenous drainage outlets of the thoracic duct: Kappa = 1.000 (almost perfect agreement); (4) Tortuosity and dilation of the thoracic segment of the thoracic duct: Kappa = 0.788 (good agreement); (5) Abnormal visualization of the bronchomediastinal trunk: Kappa = 0.482 (moderate agreement); (6) Dilation of the iliac lymphatic vessels and lumbar lymphatic trunks: Kappa = 0.256 (fair agreement).

The incidences of abnormal visualization of the subclavian trunk detected by NCMRL and ^99^TC^m^-DX lymphoscintigraphy were 18.6% (8/43) and 4.7% (2/43), respectively, with a statistically significant difference (*P* < 0.05) (Table 5).

**Table 5:** Comparison of NCMRL and ^99^TC^m^-DX lymphoscintigraphy features in 43 SLE/RA patients with lymphatic system involvement

Imaging features	NCMRL(*n* = 43)	^99^TC^m^-DX lymphoscintigraphy(*n* = 43)	Kappa value	*P* value
Obstruction of the thoracic duct outlet at the left jugular venous angle	29(67.4)	29(67.4)	1.000	1.000
Right jugular venous angle drainage of the thoracic duct and obstruction of the outlet	1(2.3)	1(2.3)	1.000	1.000
Bilateral jugular venous angle drainage of the thoracic duct and obstruction of the outlet	1(2.3)	1(2.3)	1.000	1.000
Tortuosity and dilation of the thoracic segment of thoracic duct	2(4.7)	3(7.0)	0.788	1.000
Abnormal visualisation of the subclavian trunk	**8(18.6)**	**2(4.7)**	**0.352**	**0.031**
Abnormal visualisation of the bronchial mediastinal trunk	3(7.0)	1(2.3)	0.482	0.500
Dilation of iliac lymphatic vessels and lumbar trunks	6(14.0)	1(2.3)	0.256	0.063

The values with statistical significance (*P* < 0.05) are displayed in bold

## Discussion

### Differences in clinical and imaging features of lymphatic system involvement between SLE and RA

The lymphatic system is composed of lymphatic vessels, lymph nodes, and lymphoid organs [21], among which the lymphatic vessels are composed of capillary lymphatic vessels, collecting lymphatic vessels, lymphatic trunks, and lymphatic vessels (thoracic and right lymphatic vessels). The most common clinical manifestations of lymphatic system diseases are lymphedema, ascites, and pleural effusion [22].In this study, ⁹⁹ᵐTc-DX lymphatic scintigraphy revealed chylous effusion and limb lymphedema in SLE and RA patients with lymphatic system involvement, findings consistent with those reported in the existing literature [23–25]. In our study, ^99^TC^m^-DX lymphoscintigraphy revealed the primary clinical manifestations of lymphatic system involvement in patients with SLE and RA, which is consistent with the findings of previous studies. Twenty-two (88.0%) SLE patients with lymphatic system involvement had chylous effusion: 12 (48.0%) had chylous pleural effusion, 1 (4.0%) had chylous abdominal effusion, 6 (24.0%) had chylous pleural effusion with peritoneal effusion, 2 (8.0%) had chylous pericardial effusion, and 1 (4.0%) had chylous pericardial effusion with pleural effusion. Four (22.2%) RA patients with lymphatic system involvement had chylous effusion: 2 (11.1%) had chylous pleural effusion, and 2 (11.1%) had chylous peritoneal effusion. All chyle test results were positive in patients with chylous effusion. The incidence of chylous effusion was significantly higher in SLE patients with lymphatic system involvement than in RA patients with lymphatic system involvement (*P* = 0.000). The primary manifestation in RA patients with lymphatic system involvement was limb lymphedema (14 cases, 77.8%). ^99^TC^m^-DX lymphoscintigraphy demonstrated abnormal abnormal radioactive distribution in the subcutaneous tissue of the affected limbs. The incidence of limb lymphedema was significantly higher in RA patients with lymphatic system involvement than in SLE patients with lymphatic system involvement (4 cases, 16.0%), with a statistically significant difference (*P* = 0.000).

### Role of thoracic duct obstruction in SLE and RA with lymphatic system involvement

The thoracic duct is the largest lymphatic vessel in the human body and collects lymphatic fluid from all parts of the body except for the upper right chest, upper right limb, and right side of the neck. The most common route of the thoracic duct is through the aortic hiatus into the chest cavity, passing between the azygos vein and the aorta, passing to the left at the T5–T7 level, then ascending to the level of C7, and finally flowing into the venous system at the left jugular venous angle (located at the intersection of the left internal jugular vein and the left subclavian vein) [26]. Obstruction of the thoracic duct outlet is an important cause of lymphatic reflux disorders. The primary indicators of thoracic duct outlet obstruction are visible collateral circulation and reflux [27]. In this study, ^99^TC^m^-DX lymphoscintigraphy demonstrated abnormal radioactive distribution in the thoracic duct at the jugular venous angle: 23 (92.0%) of SLE patients with lymphatic system involvement and 6 (33.3%) of the RA patients with lymphatic system involvement showed type I (abnormal concentration of left jugular venous corner), and 2 (8.0%) of the SLE patients with lymphatic system involvement showed type II (ectopic drainage and obstruction of the thoracic duct outlet), including drainage of the thoracic duct into the bilateral and right jugular venous angle. NCMRL revealed that 23 (92.0%) of the SLE patients with lymphatic system involvement and 6 (33.3%) of the RA patients with lymphatic system involvement presented with multiple abnormal lymphatic vessels in the left jugular venous angle of the thoracic duct. 1 (4.0%) of SLE with lymphatic system involvement presented with multiple abnormal lymphatic vessels at the right jugular venous angle of the thoracic duct., 1 (4.0%) of SLE with lymphatic system involvement presented with multiple abnormal lymphatic vessels at the bilateral jugular venous angles ,2 (8.0%) of SLE with lymphatic system involvement presented with tortuous dilation of the thoracic segment of the thoracic duct, 7 (28.0%) of SLE with lymphatic system involvement and 1 (5.6%) of RA with lymphatic system involvement showed abnormal display of the subclavian trunk, 3 (12.0%) of SLE with lymphatic system involvement showed abnormal visualization of the mediastinal trunk of the trachea, 5 (20.0%) of SLE with lymphatic system involvement and 1 (5.6%) of RA with lymphatic system involvement showed dilation of the iliac lymphatic vessels and lumbar trunks.These abnormal manifestations indicate that the normal lymphatic drainage pathway was obstructed, with obstruction occurring at the thoracic duct outlet. Song et al. reported 4 cases of SLE with chylous pleural effusion and found that all patients had mechanical obstruction of the thoracic duct [28]. In this study, during the exploration of thoracic duct surgery, surgeons observed that the thoracic duct in the jugular venous angle region was extensively adhered and compressed by dense fibrous tissue surrounding it, with some thickening of the duct walls; the lumen of the thoracic duct showed collapse, and the pathway for lymphatic fluid to return to the bloodstream at thoracic duct outlet was obstructed. Based on these findings, we speculate that chronic inflammation of the thoracic duct and the formation of dense fibrous adhesions with surrounding structures lead to luminal stenosis or even occlusion of the thoracic duct, resulting in obstruction at thoracic duct outlet. Persistent or severe obstruction of the thoracic duct outlet may further cause abnormal drainage, collateral formation, and dilation of lymphatic vessels throughout the lymphatic system. This could be a pathogenic basis for lymphatic drainage dysfunction-related clinical manifestations in SLE patients with lymphatic involvement and a subset of such RA patients.

### Diagnostic Value Between NCMRL and ^99^TC^m^-DX Lymphoscintigraphy

The main examination methods for lymphatic imaging include ^99^TC^m^-DX lymphoscintigraphy, direct lymphangiography, and magnetic resonance imaging [22]. The results of this study indicate that the combined application of NCMRL and ^99^TC^m^-DX lymphoscintigraphy provides valuable support for diagnosing systemic lymphatic vessel abnormalities in SLE and RA patients with lymphatic system involvement. NCMRL and ^99^TC^m^-DX lymphoscintigraphy show almost perfect agreement in demonstrating obstruction at the thoracic duct outlet (left jugular venous angle), obstruction at the right ugular vein venous drainage outlet of the thoracic duct, and obstruction at the bilateral jugular vein venous drainage outlet of the thoracic duct. The kappa values are 1.000, 1.000, and 1.000, respectively, indicating that NCMRL and ^99^TC^m^-DX lymphoscintigraphy have high concordance in evaluating the drainage direction and obstruction of the thoracic duct outlet. There was no statistically significant difference (*P* > 0.05) in the detection rates between NCMRL and ^99^TC^m^-DX lymphoscintigraphy in diagnosing tortuosity and dilation of the thoracic segment of the thoracic duct, abnormal visualisation of the bronchial mediastinal trunk, dilation of iliac lymphatic vessels and lumbar trunks; the kappa values were 0.788, 0.482, and 0.256, respectively, with good, moderate, and fair agreement, respectively. In detecting abnormal visualisation of the subclavian trunk, NCMRL was superior to ^99^TC^m^-DX lymphoscintigraphy (*P* < 0.05), which may be attributed to the relatively low spatiall resolution of ^99^TC^m^-DX lymphoscintigraphy, NCMRL is more sensitive in displaying small lymphatic vessels. Therefore, NCMRL had a higher detection rate for subclavian trunk abnormalities, which is consistent with the findings of Hao et al. [29]. Compared with NCMRL, ^99^TC^m^-DX lymphoscintigraphy can provide dynamic information. Through the abnormal movement of ^99^TC^m^-DX as a radioactive tracer in the lymphatic system, lymphedema, chyle leakage, and other conditions in SLE and RA patients with lymphatic system involvement can be visually observed. In contrast, NCMRL is a static imaging technique that lacks dynamic information, NCMRL also demonstrates serosal fluid accumulation, it cannot determine whether the fluid is chylous or localize areas of active chyle leakage.

### Exploration of pathogenic mechanisms underlying SLE and RA with lymphatic system involvement

The pathogenesis of lymphatic system involvement in SLE and RA is not fully understood. There are speculations regarding the pathogenesis of SLE combined with lymphatic system involvement: chronic inflammation of lymphatic vessels can lead to lymphatic vessel stenosis or obstruction, increased luminal pressure and increased vascular wall permeability [18], causing chylomicrons in the blood to leak out of the bloodstream and form chylous effusion. In this study, 25 SLE patients (100.0%) had thoracic duct outlet obstruction, Which provides support for the plausibility of the above speculation. A pathological feature of SLE is the activation of complement by immune complexes deposited in blood vessels, leading to vasculitis and increased vascular permeability [30]. Chylocytes in the blood directly enter the chest, abdominal, or pericardial cavities, resulting in chylous effusion. In addition, mesenteric and intestinal vasculities can increase the permeability of intestinal blood vessels to proteins [31], leading to the leakage of albumin into the intestinal lumen and the occurrence of protein-losing enteropathy. In this study, 5 (20.0%) SLE patients with lymphatic system involvement were diagnosed with protein-losing enteropathy by ^99^TC^m^-labelled human serum albumin scintigraphy, 5 patients showed mesenteric swelling on MR images, and 3 (12.0%) showed diffuse thickening of the small intestine wall.

RA can cause changes in lymph node and lymphatic vessel function. During the pathogenesis of inflammatory arthritis, changes in lymphatic vessel function include an initial compensatory expansion stage, in which the draining lymph nodes increase in size, and the lymphatic contraction and clearance of inflammation function are good. Subsequently, the lymphatic vessels enter a collapse stage, during which the local lymphatic system ruptures, and lymph node volume decreases, leading to a reduction or even loss of lymphatic return function. This results in lymphatic return dysfunction in the surrounding tissues of the joint, further exacerbating the inflammatory response. In the collapse stage, Bin cells in the draining lymph nodes migrate from the lymph node follicles to the lymphatic sinuses, where they accumulate and obstruct the lymphatic vessels [12, 32]. In this study, 9 (50%) RA patients with lymphatic system involvement had non-lymphatic reflux in the lymphedematous limb as shown by ^99^TC^m^-DX lymphoscintigraphy: the ^99^TC^m^-DX imaging agent was retained at the injection site, and the lymphatic vessels and nodes on the lymphedema side were not visualized. The remaining lymphedematous limbs showed delayed lymphatic reflux: six hours after injection, imaging revealed radioactive retention in the lymphatic vessels of the limbs, and the number of lymph node chains was normal, reduced, or poorly visualized. McRorie et al. proposed that patients with RA complicated by lymphedema may have congenital mild lymphatic vessel abnormalities, when subjected to stress factors such as polyarthritis, these potential abnormalities may manifest, leading to clinical symptoms of lymphatic obstruction and impaired drainage function [33]. In 9 (50%) RA patients with lymphatic system involvement, the lymphedematous limb demonstrated a non-lymphatic reflux pattern, and the lymphatic vessels on that side of the patient may have underlying congenital developmental abnormalities. Persistent inflammatory stimulation in RA may further damage the lymphatic vessel structure of that side of the limb, causing lymphatic vessel stenosis or even occlusion, which in turn leads to lymphatic reflux disorders and lymphedema. The above viewpoint also provides support the speculation by Schwartz et al.’s [12] that lymphatic vessels play an important role in immune tolerance while clearing fluid and inflammatory cells from inflamed tissues. When the structure or function of lymphatic vessels is abnormal and leads to lymphatic dysfunction, they may participate in the pathological and physiological processes of autoimmune diseases such as SLE and RA.

## Conclusions

In summary, distinct differences exist in the primary clinical manifestations between SLE and RA patients with SLE and RA with lymphatic system involvement: 88.0% (22/25) of SLE patients present with chylous effusion, whereas 77.8% (14/18) of RA patients manifest limb lymphedema. ^99^TC^m^-DX lymphoscintigraphy can not only display the anatomical distribution of the lymphatic system but also dynamically reflect lymphatic fluid reflux. It is a safe, non-invasive, and easy-to-operate imaging method, whereas NCMRL can clearly display the abnormal locations of lymphatic vessels and surrounding tissue conditions throughout the body. The combined application of the two examination methods provides useful value for the diagnosis of SLE and RA with lymphatic system involvement, as well as for exploring their pathogenesis.

## Study limitations and future perspectives

There are some limitations in our study. First, lymphatic system involvement in patients with SLE and RA is a rare complication, and the sample size of this study was relatively small. Despite this limitation, systematic research on this topic remains scarce to date. The findings of the present study may serve as a reference for future large-sample studies and thus hold certain exploratory value. Second, this study only enrolled patients with lymphatic system involvement diagnosed with two subtypes of connective tissue diseases: SLE and RA, it does not cover other prevalent subtypes of connective tissue diseases such as Sjogren’s syndrome and systemic sclerosis. Future research may expand the scope of inclusion to include more subtypes of patients with lymphatic system involvement, in order to comprehensively explore the clinical and imaging features of this type of disease. Besides, this study employed only two examination methods (NCMRL and ^99^TC^m^-DX lymphoscintigraphy), for evaluating lymphatic system involvement. In future research, complementary imaging techniques including ultrasonography and computed tomography lymphangiography could be further incorporated. The combined application of multimodal imaging for comprehensive assessment is expected to enhance the accuracy and reliability of both diagnostic conclusions and research findings.

## Data Availability

The datasets used and/or analyzed during the current study are available from the corresponding author on reasonable request.

## Abbreviations

SLE: Systemic lupus erythematosus
RA: Rheumatoid arthritis
NCMRL: Non-contrast Magnetic resonance lymphangiography
